# Intracardiac Echocardiography in Structural Heart Interventions: A Comprehensive Overview

**DOI:** 10.3390/jcm15030926

**Published:** 2026-01-23

**Authors:** Francesco Leuzzi, Ciro Formisano, Enrico Cerrato, Antongiulio Maione, Tiziana Attisano, Francesco Meucci, Michele Ciccarelli, Carmine Vecchione, Gennaro Galasso, Francesca Maria Di Muro

**Affiliations:** 1 Department of Medicine, Surgery, and Dentistry, University of Salerno, 84084 Salerno, Italy; f.leuzzi1@studenti.unisa.it (F.L.); c.formisano5@studenti.unisa.it (C.F.); mciccarelli@unisa.it (M.C.); cvecchione@unisa.it (C.V.); 2 Interventional Cardiology Unit, San Luigi Gonzaga University Hospital, 10043 Orbassano, Italy; enrico.cerrato@gmail.com; 3 Cardiology Unit, Cardiovascular and Thoracic Department, University Hospital “San Giovanni di Dio e Ruggi d’Aragona”, 84131 Salerno, Italy; antongiulio.maione@sangiovannieruggi.it (A.M.); tiziana.attisano@sangiovannieruggi.it (T.A.); 4 Department of Structural Interventional Cardiology, Careggi University Hospital, 50134 Florence, Italy; meuccif@aou-careggi.toscana.it; 5 Vascular Physiopathology Unit, IRCCS Neuromed Mediterranean Neurological Institute, 86077 Pozzilli, Italy

**Keywords:** intracardiac echocardiography, structural heart disease, procedural planning, procedure

## Abstract

Intracardiac echocardiography (ICE) is increasingly recognized as a valuable imaging modality in structural heart interventions, offering high-resolution, real-time visualization from within the cardiac chambers. Originally developed for electrophysiologic procedures, ICE has expanded its use across a broad spectrum of structural interventions, including atrial septal defect (ASD) and patent foramen ovale (PFO) closure, left atrial appendage occlusion (LAAO), transseptal puncture guidance, transcatheter edge-to-edge repair (TEER), balloon mitral valvuloplasty, and both mitral and tricuspid valve therapies. This review outlines the current role and technical principles of ICE, with an emphasis on catheter design, image acquisition protocols, and the emerging potential of 3D ICE. Comparisons with transesophageal echocardiography (TEE) and fluoroscopy are discussed, highlighting ICE’s ability to support minimally invasive, sedation-sparing procedures while maintaining procedural precision. We provide a focused analysis of ICE-guided applications in specific clinical scenarios, emphasizing its role in anatomical assessment, device navigation, and intra-procedural monitoring. Data from recent clinical studies and registries are reviewed to assess safety, feasibility, and outcomes. Practical considerations including operator learning curve, workflow integration, and limitations such as cost and field of view are also addressed. Lastly, we explore future directions including advanced 3D imaging, fusion imaging, artificial intelligence integration, and robotic catheter systems.

## 1. Introduction

Over the past two decades, interventional cardiology has expanded far beyond coronary revascularization to include a growing spectrum of structural heart procedures [1]. The closure of interatrial communications, left atrial appendage occlusion, and transcatheter valvular repair or replacement have progressively entered routine practice, offering less invasive options even for patients at high surgical risk [2,3]. These advances have been driven by continuous refinement in device technology, operator expertise, and—most importantly—imaging guidance [4].

Transesophageal echocardiography (TEE) has traditionally served as the reference standard for intraprocedural imaging, offering excellent spatial resolution and three-dimensional visualization of cardiac structures [5,6]. However, its reliance on deep sedation or general anesthesia limits its applicability in frail or elderly patients and introduces procedural risks, including airway compromise and esophageal injury [7]. In contrast, intracardiac echocardiography (ICE) provides high-resolution, real-time imaging directly from within the cardiac chambers, overcoming the need for esophageal intubation. The technique enhances procedural safety, patient comfort, and workflow efficiency, and is increasingly adopted for a wide range of structural interventions [8].

This review provides an updated and comprehensive overview of ICE, focusing on its technical characteristics, clinical applications, and evolving perspectives. We compare the strengths and limitations of ICE with those of conventional echocardiographic modalities and discuss emerging developments—including three-dimensional (3D) imaging, fusion technologies, and artificial intelligence—that may further expand its role in interventional cardiology. To address these objectives, the manuscript was designed as a narrative review. Evidence was identified through a targeted literature search of the PubMed database, primarily focusing on studies published within the past 10 years; earlier seminal publications were also included when relevant to the development of the field. Search terms included combinations of “intracardiac echocardiography”, “ICE-guided procedures”, “structural heart interventions”, “atrial septal defect closure”, “patent foramen ovale”, and “left atrial appendage occlusion”. Only articles published in English were considered. Eligible sources comprised original clinical studies, observational registries, narrative and systematic reviews, consensus documents, and international guideline statements. Study selection was based on relevance to the clinical use of ICE and its impact on procedural guidance and outcomes.

## 2. Technical Principles of Intracardiac Echocardiography

ICE employs a steerable ultrasound catheter, typically introduced via femoral venous access, to provide real-time, high-resolution images from within the heart [9]. Modern catheters use phased-array transducers operating at frequencies between 5 and 10 MHz, balancing axial resolution with adequate tissue penetration to visualize atrial, ventricular, and valvular structures with precision [10].

Two main technologies exist:

- **Mechanical (rotational) ICE**, now largely obsolete, produced 360° radial images with limited steerability and was primarily used in electrophysiological procedures [8,11].
- **Phased-array ICE**, the current standard, offers steerable sectorial imaging with color and spectral Doppler capabilities, enabling precise anatomical assessment and procedural guidance.

**Figure 1:**
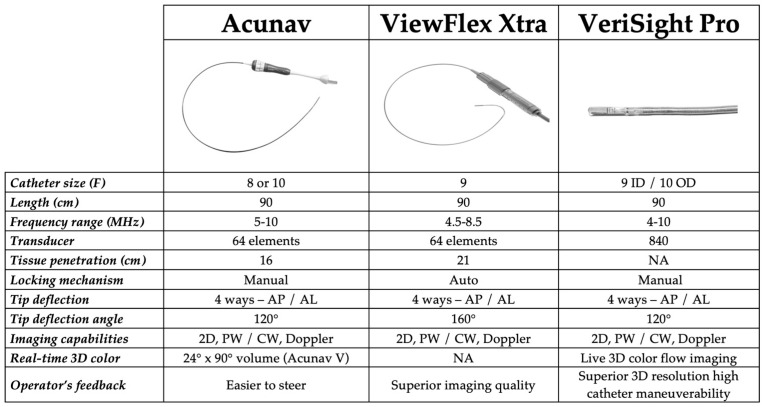
Key Comparison of commercially available ICE platforms. Abbreviations: 2D: two-dimensional; 3D: three–dimensional; AL: anterior–lateral; AP: anterior–posterior; CW: continuous wave; PW: pulsed wave.

Contemporary ICE systems, depicted in Figure 1, (e.g., AcuNav™, ViewFlex™, VeriSight Pro™) allow bidirectional deflection and integration with modern imaging consoles, providing multiplanar visualization, Doppler analysis, and, in newer platforms, real-time 3D volumetric imaging [12].

The advent of 3D and 4D ICE has markedly enhanced spatial understanding of complex anatomies and device–tissue interactions [13,14]. Matrix array transducers now permit real-time volumetric rendering and multiplanar reconstruction, offering near-TEE-quality imaging. In appropriately selected patients and institutional settings, this may facilitate procedural guidance with reduced reliance on general anesthesia, while maintaining adequate imaging quality.

## 3. Limitations, Learning Curves, and Operational Considerations

While ICE offers multiple procedural advantages, several technical and operational limitations must be acknowledged (Table 1). Compared with TEE, the field of view is narrower and far-field resolution remains suboptimal, which may restrict visualization of distant or complex structures. In selected anatomies, integration with preprocedural CT or intra-procedural fluoroscopy may be required to ensure comprehensive spatial assessment. ICE also necessitates an additional femoral venous access, with a small but non-negligible risk of vascular complications [15] that is nowadays minimized, systematically performing an echo-guided puncture even for veins.

The learning curve for ICE may be steep during the initial phase, emphasizing the need for proctored training and standardized protocols. According to the 2019 ACC/AHA/ASE Advanced Training Statement on Echocardiography, a minimum of 10 supervised ICE procedures focused on catheter manipulation and image acquisition is recommended to achieve basic competence in ICE imaging [16]. Additional experience and higher procedural volumes are required for independent use of ICE to guide complex structural heart interventions, depending on procedural complexity and institutional practice. Interventional cardiologists typically master catheter manipulation more rapidly, whereas imaging specialists often develop interpretive expertise earlier. A hybrid training model that combines these complementary skill sets facilitates earlier operator autonomy and enhances procedural consistency. Early in the learning phase, procedural times may be longer and minor pericardial effusions slightly more frequent, though these differences diminish rapidly with experience. Standardization of imaging planes and the use of structured techniques—such as double-wire or balloon-assisted septal crossing—further shorten the learning phase and improve reproducibility [17].

In selected patients, ICE may reduce the need for general anesthesia and esophageal intubation, potentially improving patient comfort and limiting anesthesia-related risks—an advantage that may be particularly relevant in elderly, frail, or pediatric populations. By providing real-time imaging, it can shorten procedure duration and reduce radiation exposure for both operators and patients [18]. Although single-use ICE catheters are relatively costly, the overall procedural cost is often balanced by shorter recovery times, simplified logistics, and greater workflow efficiency [19]. Logistics is usually simplified since ICE is generally performed directly by interventional cardiologist already involved in the procedure reducing the need of an imaging specialist in most cases. In some institution, in case of ICE utilization anesthesiologist are not required to perform LAAO/ASD/PFO.

Complications directly attributable to ICE are rare and usually minor, including transient arrhythmias, access site issues, or minimal pericardial effusions. Severe events such as tamponade or embolism are exceedingly uncommon and typically related to procedural rather than imaging factors. Routine intra-procedural ICE surveillance—including a final sweep of the pericardium and color Doppler evaluation—is recommended to ensure procedural safety [20,21].

Despite its inherent limitations and costs, ICE has become an essential imaging tool for structural heart interventions, providing real-time visualization, procedural control, and enhanced patient tolerability. Continued refinement of operator expertise and catheter technology will further consolidate its role in contemporary interventional cardiology.

## 4. Clinical Applications

### 4.1. Transseptal Puncture

ICE has become a cornerstone imaging technique for transseptal puncture (TSP), allowing precise localization of the fossa ovalis and real-time visualization of both the needle and sheath throughout the procedure [8,22].

**Figure 2:**
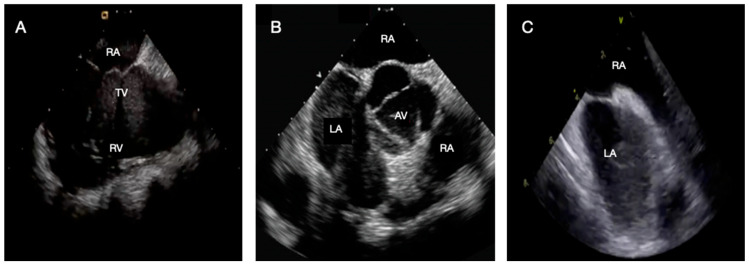
Principal ICE views from the right atrium. (**A**) Home view; (**B**) Bicaval view; (**C**) Aortic short-axis view. Abbreviation: ICE: intracardiac echocardiography; AV: aortic valve; LA: left atrium; RA: right atrium; RV: right ventricle; TV: tricuspid valve.

After insertion through an 8–10 Fr sheath, the catheter is advanced into the right atrium (RA), from which several fundamental imaging views (Figure 2) are obtained:

- **Home view**: visualization of the tricuspid valve and right ventricle.
- **Bicaval view**: alignment of the superior and inferior venae cavae and the interatrial septum (IAS).
- **Aortic short-axis view**: delineation of the aortic root in relation to the IAS.
- **Left ventricular outflow tract (LVOT) view**: comprehensive assessment of the left ventricle.

Once obtained, the bicaval view enables accurate identification of the optimal puncture site—posteroinferior for left atrial appendage occlusion and posterosuperior for transcatheter edge-to-edge repair—as recommended by current expert consensus [23].

**Figure 3:**
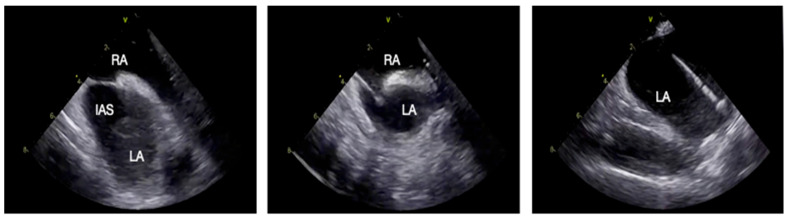
ICE-guided transseptal puncture. From left to right: visualization of the IAS; tenting of the IAS; TSP sheath in LA. Abbreviations: IAS: interatrial septum; LA: left atrium; RA: right atrium; TSP: transseptal puncture.

Once the ideal position is confirmed, the needle is advanced under direct ICE guidance, ensuring controlled puncture and safe sheath advancement into the left atrium. The sequential steps of the ICE-guided transseptal puncture are summarized in Figure 3.

Although ICE-guided transseptal puncture ensures high anatomic definition, procedural precision, and overall safety, complications may still occur—albeit infrequently (≈1% overall; mortality ≈0.15%)—and must be promptly recognized and managed [24]. The most common adverse events include the following:

- Cardiac tamponade (0.2–1.5%; up to 3% in anticoagulated patients), typically from excessive needle advancement beyond the septum; early recognition, pericardial drainage, and anticoagulation reversal are required, with sheath retention until hemostasis is achieved [25,26].
- Aortic puncture (≈0.05%), rare but potentially fatal, is more frequent in atypical anatomy or when imaging resolution is suboptimal; management ranges from observation to emergent surgical repair [27,28].
- Embolic events are clinically uncommon but have been reported as asymptomatic (“silent”) cerebral lesions detected by MRI, primarily in patients undergoing pulmonary vein isolation for atrial fibrillation ablation, with an incidence of approximately 7–13% [29,30]; These events are generally considered related to transseptal access, which represents a common procedural step across multiple left-sided interventions; however, robust incidence data outside the atrial fibrillation ablation setting are currently lacking. Adequate anticoagulation is mandatory, while the benefit of cerebral protection devices remains uncertain [31,32].
- Iatrogenic atrial septal defects, frequent in the early post-procedural phase (≤70%), usually close spontaneously; percutaneous closure is reserved for large or hemodynamically significant shunts.

### 4.2. Atrial Septal Defect and Patent Foramen Ovale Closure

Once TSP has been obtained—or deemed unnecessary, as in PFO and many secundum ASD closures—ICE becomes central to all subsequent procedural steps [33]. From the mid-RA, it provides high-resolution imaging of the IAS, allowing detailed assessment of tunnel morphology, septal thickness, fenestrations, and aneurysmal or redundant tissue. Anatomical variants such as long tunnels (>8 mm), fenestrated septa, or prominent Eustachian valves and Chiari networks can be identified beforehand, facilitating procedural planning and device selection [34].

Standard imaging planes include the septal (long-axis) and aortic (short-axis) views. The septal view, obtained by posterior catheter deflection, displays the fossa ovalis and septal rims, whereas the short-axis view—achieved by clockwise rotation and mild lateral tilt—delineates the aortic root in relation to the IAS. Additional projections provide complementary information on septal orientation and device alignment during deployment. Multiplanar and color Doppler imaging enable dynamic visualization of septal tenting, disk expansion, and device–tissue interaction, ensuring accurate positioning and stability [35].

**Figure 4:**
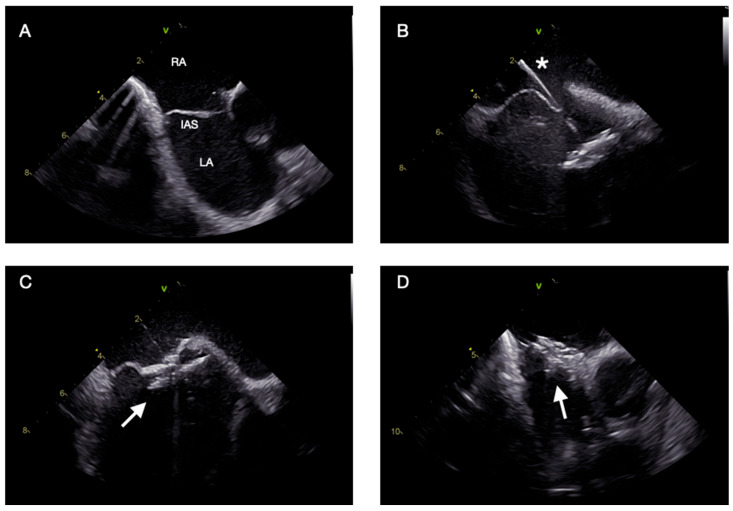
ICE-guided PFO closure: procedural steps. (**A**) Short-axis view from the right atrium showing an aneurysmal interatrial septum consistent with a PFO. (**B**) Deployment catheter (asterisk *) advanced across the PFO with the device positioned in the left atrium. (**C**) Device aligned (white arrow) but not yet released in the bicaval view. (**D**) Final appearance of the device (white arrow) after complete release. Abbreviations: ICE: intracardiac echocardiography, PFO: patent foramen ovale; RA: right atrium; IAS: interatrial septum; LA: left atrium.

Under combined ICE and fluoroscopic guidance, the PFO is crossed with a diagnostic catheter and wire, which is exchanged for a stiff wire advanced into the left superior pulmonary vein to support delivery sheath advancement [36]. The de-aired sheath is then advanced into the LA, and the occluder is sequentially deployed—first the left atrial disk, retracted gently against the septum, then the right disk to achieve full apposition and coaxial alignment across the tunnel. Final verification is performed using 2D and color Doppler ICE imaging in both long- and short-axis planes to confirm position, stability, and sealing. Agitated saline contrast testing may be used to exclude residual shunting, followed by a final ICE sweep to rule out pericardial effusion or device-related complications [37]. The most critical procedural steps and imaging planes are shown in Figure 4.

Clinical data consistently show that ICE-guided PFO closure achieves procedural success and safety comparable to TEE guidance. Its ability to integrate real-time anatomical definition with device guidance makes it particularly advantageous in complex septal anatomies or when sedation should be minimized [38,39].

### 4.3. Left Atrial Appendage Occlusion (LAAO)

LAAO is one of the most technically demanding yet rapidly evolving ICE-guided interventions. In patients with non-valvular AF and contraindications to long-term anticoagulation [40], percutaneous LAAO has become an established alternative for stroke prevention [41,42]. Although TEE remains the reference imaging modality, the increasing experience with ICE has reshaped practice, allowing high-quality intraprocedural imaging, minimizing reliance on general anesthesia or deep sedation in selected patients [43].

From the RA, ICE enables precise localization of the fossa ovalis and real-time TSP guidance—typically in a postero-inferior position to ensure coaxial access to the LAA—while allowing early recognition of potential complications. Once LA access is achieved, ICE provides a detailed visualization of LAA morphology and its relationship with adjacent structures, including the mitral annulus, pulmonary veins, and Coumadin ridge [44]. Because direct visualization of the LAA from the RA is limited—particularly for accurate ostial sizing, landing-zone assessment, and thrombus exclusion—most operators advance the ICE catheter into the LA through the same TSP used for device delivery, obtaining stable, high-resolution imaging of the appendage and its anatomical boundaries.

Crossing the IAS with the ICE catheter requires experience and careful manipulation but can be safely performed using standardized approaches [45]:

- **Probing technique**: advancing the catheter along a guidewire positioned in a pulmonary vein through the dilated IAS [46].
- **Double-wire technique**: using a stiff support wire (e.g., Safari or Amplatz) to straighten the septal tract and facilitate crossing.
- **Snaring technique**: advancing a gooseneck snare from the RA to capture and guide the ICE tip through the puncture site [47].
- **Balloon dilation**: pre-dilating the TSP with an 8 mm balloon to ease crossing, with no increase in iatrogenic ASD rates [48].

Once positioned in the LA, ICE offers multiple acoustic windows analogous to TEE views:

- **LA home view**: from the mid-LA with mild posterior deflection and counterclockwise rotation, visualizing the mitral annulus and LAA ostium for ostial and landing-zone measurements.
- **Left Superior Pulmonary Vein (LSPV) view**: by advancing the catheter into the left superior pulmonary vein and rotating toward the appendage, providing a coaxial long-axis perspective ideal for depth assessment and monitoring device expansion during deployment [49].
- **Inferior (mitral) view**: by advancing slightly toward the basal LV and facing the appendage from below, allowing en-face visualization of device compression, sealing, and relation to the mitral apparatus [50].

Additional projections from the left inferior pulmonary vein or atrial roof can assist in complex multilobar or angulated anatomies.

**Figure 5:**
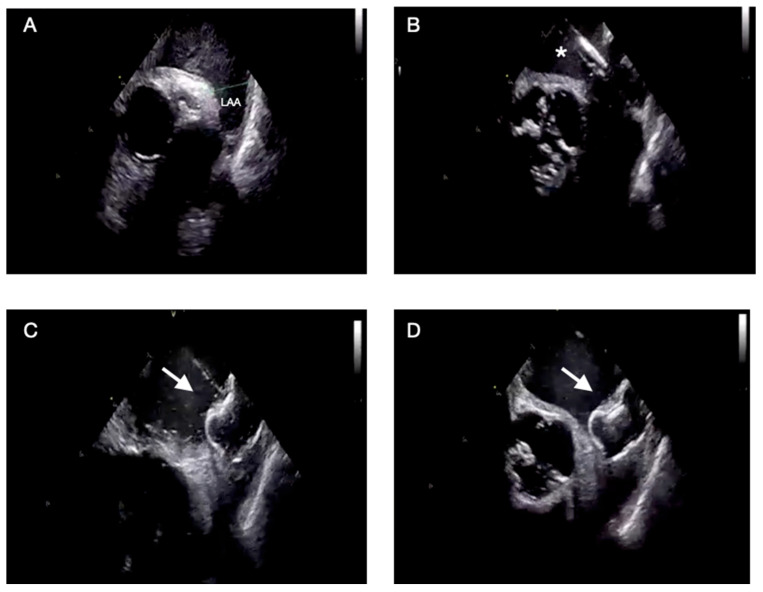
ICE-guided LAAO: procedural steps. (**A**) LSPV view with measurement of LAA dimensions to guide device sizing. (**B**) Delivery catheter (asterisk *) advanced into the LAA under ICE guidance in the left-atrial “home view.” (**C**) Partial deployment of the Occluder device (white arrow), still attached to the delivery cable. (**D**) Final device release (white arrow) with confirmation of stability and appropriate sealing of the ostial margins. Abbreviations: ICE: intracardiac echocardiography; LAA: left atrial appendage; LAAO: left atrial appendage occlusion; LSPV: left superior pulmonary vein.

Under combined fluoroscopic and ICE guidance, the delivery sheath is aligned with the appendage axis, and the occlusion device is advanced into the target position. The anchoring or occluding component (lobe or plug) is deployed at the pre-determined depth within the LAA, with real-time confirmation of coaxial alignment, anchoring, and compression according to manufacturer-specific criteria [51]. When present, an ostial sealing element (such as a disk) is subsequently released to ensure complete apposition and closure. Before final release, the PASS (Position, Anchoring, Size, Seal) criteria are systematically verified using 2D and color Doppler imaging to confirm device stability, adequate compression, and effective sealing, while excluding interference with the mitral valve, pulmonary veins, or atrial wall [42]. Furthermore, a systematic final ICE sweep of the LA is performed to rule out pericardial effusion, device malalignment, or embolization. Agitated saline contrast testing or a contrast injection confirms complete closure and absence of residual shunt. Representative procedural steps and imaging planes are shown in Figure 5.

Observational and prospective studies have confirmed the feasibility and safety of ICE-guided LAAO. In the multicenter ICE-LAA study [52], technical success reached 100% with no conversions to TEE and no excess in major adverse events compared with the PINNACLE-FLX trial [53], where TEE was the primary imaging modality. Procedural and fluoroscopy times were comparable, while total in-room and recovery times were significantly shorter with ICE. The rates of peri-device leaks ≤ 5 mm, pericardial effusion, and iatrogenic ASD were also similar. Importantly, ICE enables reliable thrombus exclusion and early detection of complications, enhancing procedural safety and workflow efficiency [54].

### 4.4. Mitral Interventions

#### 4.4.1. Mitral Transcatheter Edge-to-Edge Repair—M-TEER

The application of ICE in M-TEER has been described primarily in isolated reports and small series, with most experiences employing advanced 3D or 4D systems capable of multiplanar and volumetric imaging [55,56,57]. Once LA access is obtained, ICE provides a detailed picture of the mitral annulus, leaflets, and subvalvular structures, supporting each procedural step from device positioning to final assessment. Through controlled deflection and rotation within the LA, the operator can reproduce the standard working planes familiar from TEE. The bicommissural view (approximately −45° to +45°) displays both commissures and the grasping area, whereas the left ventricular outflow tract (LVOT) view (+45° to +135°) offers a sagittal perspective including the mitral inflow and left ventricular cavity. These projections allow continuous assessment of clip orientation, leaflet capture, and residual MR, while multiplanar reconstruction facilitates evaluation of spatial alignment and device–leaflet interaction [58]. Color Doppler imaging quantifies post-repair MR and pulmonary vein flow patterns, and transmitral gradients are readily measured in real time. A final imaging survey ensures adequate coaptation, absence of subvalvular interference, and confirmation of stable device position. Although the shared transseptal tract between ICE and the delivery system may occasionally limit maneuverability or cause acoustic shadowing, high-quality anatomical information can be achieved with optimized probe orientation and gentle torque adjustments [59].

While large-scale validation is still lacking, initial experiences demonstrate the feasibility of ICE-guided M-TEER, particularly with next-generation 3D and 4D catheters offering enhanced spatial resolution and wider imaging sectors [60]. Integration of ICE data with fluoroscopy or fusion platforms may further refine procedural control, promoting a complementary rather than substitutive role alongside TEE in complex mitral interventions [61].

#### 4.4.2. Transcatheter Mitral Valve Replacement (TMVR)

ICE has also been applied in TMVR, particularly for valve-in-valve or valve-in-ring procedures and in patients unsuitable for TEE [62]. Although the experience remains limited to case reports and small series, these studies demonstrate the feasibility of using ICE as the sole imaging modality for percutaneous mitral bio-prosthesis implantation [63,64].

When positioned in the LA, ICE allows direct visualization of the mitral annulus, prosthetic frame, and sub-valvular structures, enabling precise anatomical characterization and real-time procedural guidance. In the context of degenerating surgical or transcatheter mitral bio-prostheses, ICE facilitates assessment of leaflet motion, valve morphology, and effective orifice area (EOA) reduction. Using 3D and multiplanar reconstruction (MPR), the dysfunctional prosthesis can be accurately evaluated before deployment, with detailed visualization of the landing zone, annular calcification, and the spatial relationship between the prosthesis and LVOT.

During implantation, volumetric ICE imaging provides continuous monitoring of valve expansion, coaxial alignment, and interaction with surrounding structures. Doppler interrogation confirms hemodynamic performance, detecting perivalvular leaks or LVOT obstruction early. These real-time insights support accurate valve positioning and help mitigate procedural complications.

Published experiences have reported successful ICE-guided TMVR in patients with contraindications to TEE, confirming the ability of ICE to visualize prosthesis seating, leaflet motion, and residual regurgitation with high fidelity [65,66]. The procedural workflow and catheter handling largely mirror those described for M-TEER, leveraging similar imaging windows and manipulation strategies [67].

Although current evidence is confined to small-scale experiences, ICE appears to offer a reliable imaging alternative when TEE is not feasible or desirable. By providing direct high-resolution views from within the LA, it enhances procedural precision, ensures immediate recognition of complications such as malalignment or pericardial effusion, and may simplify intraprocedural coordination by reducing dependency on a second imaging operator [68].

Future developments in 3D and 4D ICE technology—offering improved spatial resolution, wider fields of view, and multimodal integration—are expected to further consolidate its role in TMVR [69]. Equally important is high-quality pre-procedural imaging, particularly cardiac CT, which remains essential for thorough annular and subvalvular assessment and for procedural planning [70]. The integration of CT-derived models with real-time ICE through fusion imaging platforms—combining CT- and echo-guided navigation—is anticipated to enhance anatomical orientation, device positioning, and overall procedural safety. As experience grows, ICE may transition from an alternative imaging option to a complementary or even primary modality for selected patients undergoing complex mitral replacement procedures.

#### 4.4.3. Percutaneous Transvenous Mitral Commissurotomy (PTMC)

ICE has also been applied to guide PTMC in patients with rheumatic mitral stenosis, particularly when TEE is contraindicated. Early clinical experiences demonstrated that ICE provides adequate visualization of the interatrial septum, LA, and LAA, supporting both procedural planning and real-time guidance throughout the intervention [71,72,73].

In a retrospective series of 20 patients, Saji et al. reported the feasibility of ICE-guided PTMC using an 8-Fr phased-array catheter [74]. The catheter, positioned in the RA and rotated clockwise, accurately delineated the fossa ovalis to guide TSP. After confirming the absence of thrombus, the ICE probe was advanced into the LA through a Mullins introducer, enabling continuous imaging during balloon valvuloplasty. All procedures were completed successfully, with no ICE-related complications, confirming image quality comparable to TEE for septal visualization and thrombus exclusion.

Similarly, Green et al. described 19 cases of ICE-guided balloon mitral valvuloplasty, in which ICE facilitated septal puncture, optimal balloon positioning, and prompt recognition of procedural complications such as severe MR [75]. In addition to procedural guidance, ICE enables accurate measurement of the mitral annulus, assisting in appropriate balloon sizing and procedural planning.

Recent advances with 3D-ICE have further improved spatial orientation and anatomical delineation of the mitral valve apparatus. Real-time biplane and volumetric imaging allow more accurate visualization of commissural splitting, subvalvular structures, and immediate post-dilation results. Early feasibility studies confirm the practicality of this approach, suggesting that volumetric ICE may refine both procedural safety and precision in PTMC [76].

### 4.5. Tricuspid Interventions

TEE imaging during T-TEER can be technically demanding because of the complex and variable tricuspid valve anatomy, leaflet orientation, and acoustic interference from adjacent right-sided structures [77,78]. In this context, ICE offers an effective alternative for procedural guidance, providing direct real-time visualization of the TV and right-sided cardiac chambers.

After obtaining the home view in the RA, the probe is oriented anteriorly toward the TV to optimize leaflet visualization and minimize shadowing. Lateral flexion and rotation along the commissural plane allow optimal alignment for biplane and 3D acquisitions. From the RA, 3D-ICE enables detailed visualization of the septal, anterior, and posterior leaflets, although spatial and temporal resolution remain slightly inferior to TEE [79].

Using 3D multiplanar reconstruction (MPR), inflow–outflow, septal–lateral, and atrial en-face views can be obtained simultaneously. Rotation of the 3D dataset around the z-axis (~90°) positions the aorta at the 5-o’clock reference, mirroring standard TEE orientation and enabling accurate localization of tricuspid regurgitation (TR) jets with color Doppler. During leaflet grasping, ICE confirms leaflet insertion and device–leaflet alignment, while fluoroscopy ensures correct orientation and minimizes parallax. After device release, 4D-ICE provides immediate confirmation of leaflet capture, coaptation, and TR reduction.

In a small series of patients undergoing T-TEER, 4D-ICE demonstrated comparable image quality and measurement accuracy to TEE, with adequate visualization of the TV apparatus and commissures [80]. However, TEE still offers superior spatial and temporal resolution for detailed anatomical definition [81]. Ongoing advances in volumetric ICE, including higher frame rates and improved Doppler sensitivity, are expected to further enhance its role in right-sided interventions.

Preliminary experiences have confirmed the feasibility and safety of ICE-guided T-TEER. Nonetheless, its systematic application in transcatheter tricuspid valve replacement (T-TVR) remains to be established. In these early reports, ICE and TEE were used in a complementary fashion, with each modality providing additional imaging support to the other throughout the procedure [82]. Future studies are warranted to determine its diagnostic accuracy and procedural utility in this setting.

**Figure 6:**
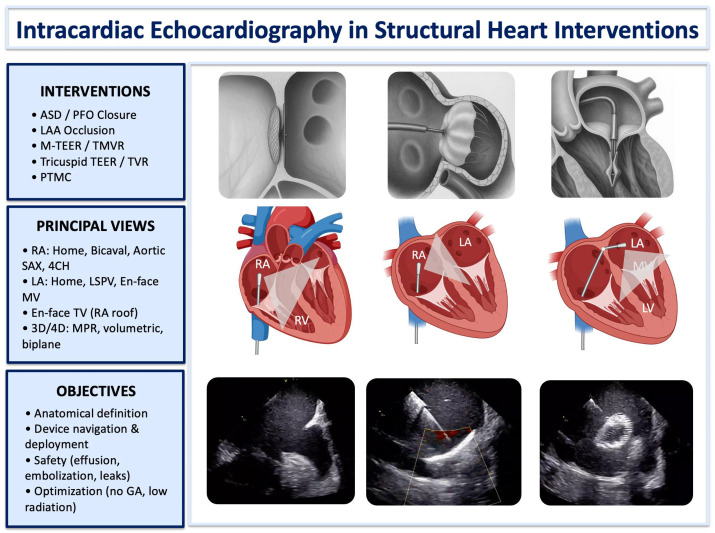
Intracardiac Echocardiography in Structural Heart Interventions. Integrated schematic overview of intracardiac echocardiography in structural heart interventions. The figure summarizes: (i) the main ICE-guided procedures (ASD/PFO closure, LAA occlusion, mitral and tricuspid edge-to-edge repair or replacement, PTMC); (ii) principal right- and left-atrial imaging views used for procedural guidance; (iii) core objectives of ICE (anatomical definition, device navigation and deployment, safety monitoring, and optimization without general anesthesia); and (iv) representative ICE images illustrating anatomic visualization and device–tissue interaction. Abbreviations: 3D/4D: three-/four-dimensional; A4CH: apical four-chamber; ASD: atrial septal defect; GA: general anesthesia; ICE: intracardiac echocardiography; LA: left atrium; LAA: left atrial appendage; LSPV: left superior pulmonary vein; LV: left ventricle; MPR: multiplanar reconstruction; MV: mitral valve; PFO: patent foramen ovale; PTMC: percutaneous transvenous mitral commissurotomy; RA: right atrium; RV: right ventricle; SAX: short-axis; TEER: transcatheter edge-to-edge repair; TV: tricuspid valve; TVR: tricuspid valve replacement.

A comprehensive overview of the main structural interventions guided by ICE, the key right- and left-atrial imaging projections, and the core intraprocedural objectives is shown in Figure 6.

## 5. Future Perspectives: 3D ICE, Fusion Imaging, Artificial Intelligence, and Robotics

The future of ICE lies in technological integration and multimodal fusion. Next-generation 3D ICE systems already enable real-time volumetric imaging with improved spatial resolution and color Doppler capability [83,84,85]. When combined with fluoroscopy or 3D rotational angiography, these platforms offer synchronized visualization of both anatomy and device motion, enhancing procedural precision.

Fusion imaging—overlaying ICE data onto fluoroscopic or CT-derived models—represents an emerging frontier, facilitating orientation, catheter navigation, and device positioning, particularly in complex transcatheter valve interventions [86].

Additionally, artificial intelligence (AI) is expected to further transform ICE imaging by automating structure recognition, optimizing probe orientation, and providing real-time guidance for less experienced operators. Early prototypes already integrate AI-based edge detection and automatic 3D reconstruction, supporting intra-procedural decision-making [87]. Lastly, robotic catheter systems are under development to improve catheter stability and fine manipulation, potentially allowing remote operation and greater reproducibility of imaging views [88]. Combined with 3D ICE and AI-assisted fusion, these advances are poised to redefine intra-procedural imaging, supporting fully image-guided, minimally invasive structural interventions with unprecedented precision and safety.

In addition, ICE application may extend beyond structural procedures in the future and represent a potential imaging modality for guiding the implantation of temporary or durable mechanical circulatory support (MCS) devices by providing real-time visualization of cardiac structures and device positioning during deployment. However, this application remains largely unexplored. Importantly, MCS implantation is frequently performed in patients with severe hemodynamic compromise or cardiogenic shock—clinical scenarios that may limit the feasibility and safety of ICE use because of time constraints, vascular access challenges, and the need for rapid device deployment. Consequently, the role of ICE in MCS implantation should currently be regarded as investigational, and dedicated studies are required to define its feasibility, safety, and incremental value compared with established imaging modalities.

## 6. Conclusions

ICE has evolved from an ancillary imaging tool in electrophysiology to a cornerstone modality in contemporary structural heart interventions. By providing high-resolution, real-time visualization of cardiac anatomy, ICE may, in selected patients and centers, reduce the need for general anesthesia or deep sedation, depending on procedural complexity, patient characteristics, and institutional workflow, without compromising procedural accuracy and with potential benefits in terms of patient safety and tolerance.

Beyond transseptal puncture and interatrial defect closure, ICE is increasingly used to guide left- and right-sided procedures, including LAA occlusion and mitral or tricuspid repair and replacement—allowing smoother procedural workflows, streamlined coordination among operators, and greater autonomy for interventional teams. Ongoing advances in three-dimensional volumetric imaging, multimodality fusion, and artificial intelligence–assisted navigation are expected to further expand ICE into a fully integrated intraprocedural imaging platform, capable of combining anatomical visualization, hemodynamic assessment, and device guidance in real time. As these technologies mature, ICE is likely to assume an increasingly central role in supporting safer and more efficient complex transcatheter interventions.

## Figures and Tables

**Table 1:** Key Advantages and Limitations of ICE in Structural Heart Interventions.

Advantages	Limitations
May allow procedures to be performed under local anesthesia with or without deep sedation, potentially avoiding general anesthesia in selected patients and centers, depending on procedural complexity and institutional protocols	Steep learning curve requiring specific training and experience
Real-time, high-resolution imaging of cardiac structures	Higher cost compared with standard fluoroscopy or TEE-guided procedures
Avoids esophageal intubation and related complications compared to TEE	Limited field of view compared to TEE, especially for posterior structures
Reduced radiation exposure for both patient and operator	Requires dedicated venous access and additional catheter manipulation
Feasibility in patients with contraindications to TEE (esophageal disease, intolerance)	Potential vascular complications at the venous access site
Facilitates immediate detection and management of procedural complications (e.g., pericardial effusion, device malposition)	Less widespread availability and operator familiarity in some centers

## Data Availability

No new data were created or analyzed in this study. Data sharing is not applicable to this article.

## Abbreviations

The following abbreviations are used in this manuscript:

	**Definition**
3D	three-dimensional
4D	four-dimensional
AF	atrial fibrillation
AI	artificial intelligence
ASD	atrial septal defect
CT	computed tomography
EOA	effective orifice area
IAS	interatrial septum
ICE	intracardiac echocardiography
LA	left atrium
LAA	left atrial appendage
LAAO	left atrial appendage occlusion
LSPV	left superior pulmonary vein
LV	left ventricle
LVOT	left ventricular outflow tract
M-TEER	mitral transcatheter edge-to-edge repair
MPR	multiplanar reconstruction
MR	mitral regurgitation
MRI	magnetic resonance imaging
PFO	patent foramen ovale
PTMC	percutaneous transvenous mitral commissurotomy
RA	right atrium
RV	right ventricle
T-TEER	transcatheter tricuspid edge-to-edge repair
T-TVR	transcatheter tricuspid valve replacement
TEE	transesophageal echocardiography
TEER	transcatheter edge-to-edge repair
TMVR	transcatheter mitral valve replacement
TR	tricuspid regurgitation
TSP	transseptal puncture
TV	tricuspid valve
